# Adding abiraterone or docetaxel to long-term hormone therapy for prostate cancer: directly randomised data from the STAMPEDE multi-arm, multi-stage platform protocol

**DOI:** 10.1093/annonc/mdy072

**Published:** 2018-02-26

**Authors:** M R Sydes, M R Spears, M D Mason, N W Clarke, D P Dearnaley, J S de Bono, G Attard, S Chowdhury, W Cross, S Gillessen, Z I Malik, R Jones, C C Parker, A W S Ritchie, J M Russell, R Millman, D Matheson, C Amos, C Gilson, A Birtle, S Brock, L Capaldi, P Chakraborti, A Choudhury, L Evans, D Ford, J Gale, S Gibbs, D C Gilbert, R Hughes, D McLaren, J F Lester, A Nikapota, J O’Sullivan, O Parikh, C Peedell, A Protheroe, S M Rudman, R Shaffer, D Sheehan, M Simms, N Srihari, R Strebel, S Sundar, S Tolan, D Tsang, M Varughese, J Wagstaff, M K B Parmar, N D James, The STAMPEDE Investigators

**Affiliations:** 1 MRC Clinical Trials Unit at UCL, London; 2 Cardiff University, Cardiff; 3 Christie and Royal Salford Hospital, Manchester; 4 Institute of Cancer Research, Sutton; 5 UCL Cancer Institute, University College London, London; 6 Guy's & St Thomas NHS, Foundation Trust, London; 7 St James University Hospital, Leeds, UK; 8 Division of Oncology and Hematology, Kantonsspital St. Gallen, St. Gallen; 9 University of Bern, Bern; 10 Swiss Group for Cancer Clinical Research (SAKK), Bern, Switzerland; 11 The Clatterbridge Cancer Centre NHS Foundation Trust, Liverpool; 12 Institute of Cancer Sciences, University of Glasgow, Glasgow; 13 Beatson West of Scotland Cancer Centre, University of Glasgow, Glasgow; 14 Royal Marsden Hospital, Sutton; 15 Faculty of Education, Health and Wellbeing, University of Wolverhampton, Wolverhampton; 16 Rosemere Cancer Centre, Royal Preston Hospital, Preston; 17 Dorset Cancer Centre, Poole Hospital, Poole; 18 Worcestershire Acute Hospitals NHS Trust, Worcester; 19 Royal Derby Hospital, Derby; 20 Division of Cancer Sciences, University of Manchester, Manchester; 21 Manchester Academic Health Science Centre, Manchester; 22 Christie Hospital NHS Foundation Trust, Manchester; 23 Sheffield Teaching Hospitals NHS Foundation Trust, Sheffield; 24 City Hospital, Cancer Centre at Queen Elizabeth Hospital, Birmingham; 25 Portsmouth Oncology Centre, Queen Alexandra Hospital, Portsmouth; 26 Queen's Hospital, Romford; 27 Sussex Cancer Centre, Royal Sussex County Hospital, Brighton; 28 Mount Vernon Group, Mount Vernon Hospital, Middlesex; 29 Western General Hospital, Edinburgh; 30 Velindre Cancer Centre, Cardiff; 31 Sussex Cancer Centre, Brighton; 32 Centre for Cancer Research and Cell Biology, Queens University Belfast, Belfast; 33 Belfast City Hospital, Belfast; 34 Lancashire Teaching Hospitals NHS Trust, Preston; 35 Department of Oncology & Radiotherapy, South Tees NHS Trust, Middlesbrough; 36 Oxford University Hospitals NHS Foundation Trust; 37 Department of Oncology, Royal Surrey County Hospital, Guildford; 38 Royal Devon and Exeter Hospital, Exeter; 39 Hull & East Yorkshire Hospitals NHS Trust, Hull; 40 Shrewsbury and Telford Hospitals NHS Trust, Shrewsbury, UK; 41 Kantonsspital Graubünden, Chur; 42 Swiss Group for Cancer Clinical Research (SAKK), Bern, Switzerland; 43 Department of Oncology, Nottingham, University Hospitals NHS Trust, Nottingham; 44 Southend Hospital, Southend-on-Sea; 45 Musgrove Park Hospital, Taunton and Somerset NHS Foundation Trust; 46 Swansea University College of Medicine, Swansea; 47 Institute of Cancer and Genomic Sciences, University of Birmingham, Edgbaston, Birmingham, UK

**Keywords:** prostate cancer, randomised, treatment, abiraterone, docetaxel, head-to-head

## Abstract

**Background:**

Adding abiraterone acetate with prednisolone (AAP) or docetaxel with prednisolone (DocP) to standard-of-care (SOC) each improved survival in systemic therapy for advanced or metastatic prostate cancer: evaluation of drug efficacy: a multi-arm multi-stage platform randomised controlled protocol recruiting patients with high-risk locally advanced or metastatic PCa starting long-term androgen deprivation therapy (ADT). The protocol provides the only direct, randomised comparative data of SOC + AAP versus SOC + DocP.

**Method:**

Recruitment to SOC + DocP and SOC + AAP overlapped November 2011 to March 2013. SOC was long-term ADT or, for most non-metastatic cases, ADT for ≥2 years and RT to the primary tumour. Stratified randomisation allocated pts 2 : 1 : 2 to SOC; SOC + docetaxel 75 mg/m^2^ 3-weekly×6 + prednisolone 10 mg daily; or SOC + abiraterone acetate 1000 mg + prednisolone 5 mg daily. AAP duration depended on stage and intent to give radical RT. The primary outcome measure was death from any cause. Analyses used Cox proportional hazards and flexible parametric models, adjusted for stratification factors. This was not a formally powered comparison. A hazard ratio (HR) <1 favours SOC + AAP, and HR > 1 favours SOC + DocP.

**Results:**

A total of 566 consenting patients were contemporaneously randomised: 189 SOC + DocP and 377 SOC + AAP. The patients, balanced by allocated treatment were: 342 (60%) M1; 429 (76%) Gleason 8–10; 449 (79%) WHO performance status 0; median age 66 years and median PSA 56 ng/ml. With median follow-up 4 years, 149 deaths were reported. For overall survival, HR = 1.16 (95% CI 0.82–1.65); failure-free survival HR = 0.51 (95% CI 0.39–0.67); progression-free survival HR = 0.65 (95% CI 0.48–0.88); metastasis-free survival HR = 0.77 (95% CI 0.57–1.03); prostate cancer-specific survival HR = 1.02 (0.70–1.49); and symptomatic skeletal events HR = 0.83 (95% CI 0.55–1.25). In the safety population, the proportion reporting ≥1 grade 3, 4 or 5 adverse events ever was 36%, 13% and 1% SOC + DocP, and 40%, 7% and 1% SOC + AAP; prevalence 11% at 1 and 2 years on both arms. Relapse treatment patterns varied by arm.

**Conclusions:**

This direct, randomised comparative analysis of two new treatment standards for hormone-naïve prostate cancer showed no evidence of a difference in overall or prostate cancer-specific survival, nor in other important outcomes such as symptomatic skeletal events. Worst toxicity grade over entire time on trial was similar but comprised different toxicities in line with the known properties of the drugs.

**Trial registration:**

Clinicaltrials.gov: NCT00268476.

Key Message

Abiraterone acetate and docetaxel, with predniso(lo)ne (AAP, DocP) separately improved survival when added to standard-of-care for hormone-sensitive prostate cancer. STAMPEDE randomised 566 patients to these treatment arms when both were accruing, the only head-to-head data available. No evidence of a difference in overall or prostate cancer-specific survival.

## Research in context

### Evidence before this study

Abiraterone acetate plus prednisone/prednisolone (AAP) and docetaxel with prednisone/prednisolone (DocP) have separately been shown to improve survival when used in addition to the previous international standard-of-care (SOC) for hormone-sensitive prostate cancer of androgen deprivation therapy with further therapy such as AAP or DocP on relapse. This has been confirmed in a number of separate trials and on meta-analysis. The largest body of evidence for both AAP and DocP comes from the systemic therapy for advanced or metastatic prostate cancer: evaluation of drug efficacy (STAMPEDE) platform trial.

### Added value of this study

Recruitment to DocP and AAP overlapped in STAMPEDE giving the only head-to-head evidence comparing these two new standard treatment approaches. We report data from the 566 patients who were directly randomised between these two treatment approaches while the two research arms were both open to recruitment. The data show strong evidence favouring SOC + AAP on earlier, more biochemically driven outcome measures (OMs). For longer-term, more clinically driven OMs, including bone complications, prostate cancer-specific and overall survival, there is no evidence of a significant difference between AAP and DocP.

### Implications of all the available evidence

The reported trials and meta-analyses showed a larger effect on survival for AAP over the previous SOC than did DocP over the standard SOC. These data show that the story may be more complicated. No other directly randomised data on survival of these treatments are available. Individual patient data network meta-analysis using all of the published trials are warranted, accounting for differences in patient characteristics, treating clinicians and centres and salvage treatment access. The STAMPEDE team is collaborating with the STOPCAP meta-analysis group to achieve this.

## Introduction

For several decades, the standard-of-care (SOC) for most patients with high-risk locally advanced or metastatic prostate cancer has been long-term androgen deprivation therapy (ADT) alone. The past few years, there have been great changes, first with results from randomised controlled trials (RCTs) showing a survival advantage compared with ADT alone for adding radiotherapy to the prostate in men with non-metastatic disease and no known nodal involvement [1–3]; then with systemic treatments for all men starting long-term hormone therapy: docetaxel plus prednisolone/prednisone (DocP) [4–9] and, most recently, abiraterone acetate plus prednisolone/prednisone (AAP) [10, 11]. As both therapeutic combinations are effective, there are now two distinct standards-of-care with little information to guide clinicians as to which is the more effective; there are no prospective, powered, RCTs that will deliver direct comparative data.

Systemic therapy for advanced or metastatic prostate cancer: evaluation of drug efficacy (STAMPEDE) is a multi-arm, multi-stage platform protocol which assessed both of these treatment approaches, separately, against the previous SOC [12, 13]. The ‘docetaxel comparison’ of STAMPEDE recruited patients allocated to SOC + DocP between October 2005 and March 2013. The ‘abiraterone comparison’, the first comparison to be added to STAMPEDE, recruited patients allocated to SOC or SOC + AAP between November 2011 and January 2014. Each of those comparisons had primary outcome measure (OM) of overall survival (OS) for the patients randomised contemporaneously to the control arm and the relevant research arm. Consequently, between 15 November 2011 and 31 March 2013, patients were directly randomised contemporaneously between these two research arms (and other research arms) and we now present these data. 

## Methods

### Trial design

The STAMPEDE protocol and design have been described in detail elsewhere [7, 10, 12, 14]. Briefly, STAMPEDE comprises a series of multi-arm multi-stage (MAMS) comparisons that have overlapped in recruitment and follow-up time. 

### Patient selection

Eligible patients were those starting long-term ADT for the first time. This was defined as patients with metastatic disease, nodal involvement or node negative, non-metastatic disease with two or more of three high-risk features: T-category 3 or 4, Gleason sum score 8–10 or PSA > 40 ng/ml. Patients rapidly relapsing after previous local therapy were also permitted if they had PSA > 20 ng/ml or PSA > 4 ng/ml with a PSA doubling time <6 months or those who developed loco-regional or metastatic spread whilst not on hormone therapy.

As with all STAMPEDE comparisons, the primary OM of the two underpinning comparisons (against control) was OS. Failure-free survival (FFS) was an intermediate primary OM, defined as time from randomisation to the first of: rising PSA (where rising PSA was defined as a confirmed rise to >4 ng/ml, and >50% above the lowest value in the first 6 months after randomisation); new disease or progression of: distant metastases, lymph nodes or local disease; or death from prostate cancer. Progression-free survival (PFS) was defined as time from randomisation to the first of: new disease or progression of: distant metastases, lymph nodes or local disease; or death from prostate cancer [15]. Metastatic PFS (MPFS) was defined as time from randomisation to death from any cause, new metastases or progression of distant metastases.

All patients provided written informed consent; all versions of the protocol have been reviewed by the relevant research ethics committees and the regulatory agencies; the original protocol and all subsequent versions involving the introduction of a new research arm and comparison were independently peer-reviewed by Cancer Research UK (CRUK).

**Figure 1.**
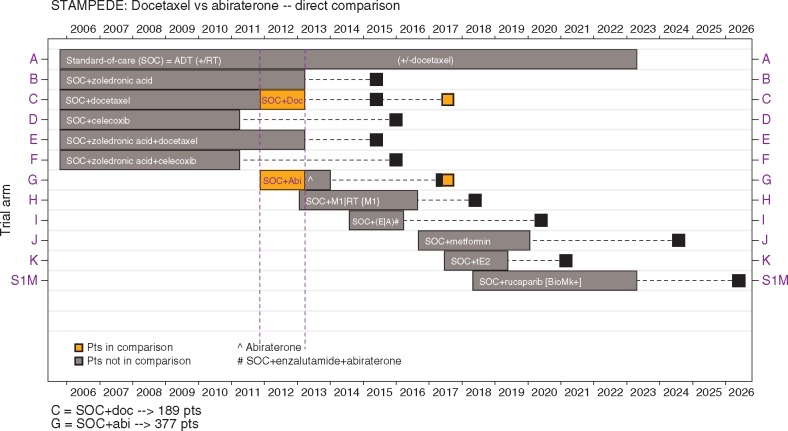
Activity-by-time diagram: patients included in this comparison. SOC, standard-of-care; Doc, docetaxel; Abi, abiraterone acetate+prednisone/prednisolone. Boxes represents periods of recruitment (*x*-axis) to each of the trial arms (*y*-axis). The blue boxes represent recruitment periods contributing to this analysis; the green boxes other recruitment period, past and future, contributing to other aspects of the STAMPEDE. The squares represent the time point of the first key comparative analyses for each comparison in pink and for this comparison in blue.

Patients have been allocated across a number of research treatments as depicted in Figure 1. Here we focus on those patients randomised between 15 November 2011 and 31 March 2013, while both the ‘docetaxel comparison’ and the ‘abiraterone comparison’ were open to recruitment, and who were allocated to either SOC + DocP or SOC + AAP.

### Trial treatment, masking and follow-up

The SOC was long-term hormone therapy with LHRH analogues (with short term antiandrogen if relevant) or orchidectomy. Unless contraindicated, radiotherapy to the prostate was mandated in all patients with N0M0 disease, encouraged in patient with N + M0 disease, and permitted in patients with M1 disease until the activation of the ‘M1|RT comparison’ in January 2013. On the DocP arm, docetaxel (75 mg/m^2^) was given once every 3 weeks for six cycles, with prednisolone/prednisone (10 mg) daily. On the AAP arm, abiraterone acetate (1000 mg) with prednisolone/prednisone (5 mg) daily was given until PSA, clinical and radiological progression or a change of treatment. AAP duration was capped after 2 years in M0 patients having radical radiotherapy. Modifications for toxicities were described in the protocol and previous papers [7, 10]. Treatment allocation was not masked for practical reasons. Patients were seen 6-weekly at first, dropping to 6-monthly after 2 years. Imaging scans after baseline were at the investigator’s discretion.

### Randomisation

Patients were randomised centrally using minimisation with a random element across a number of stratification factors using unequal allocation (previously described) [7, 10]. The allocation ratio was initially 2 : 1 control : research; the ‘abiraterone comparison’ was brought in with an equal allocation (1 : 1) ratio to the control. Therefore the allocation ratio here is 1 : 2 for SOC + DocP : SOC + AAP.

### Statistical analysis

The comparison presented here is of SOC + AAP against SOC + DocP because both of these arms have demonstrated better OS than their contemporaneous controls in the population of men starting long-term hormone therapy. The protocol specified that research arms which were better than the control arm could be compared, following a closed test approach. The maturity of the data used for SOC + AAP matches that recently reported [10] in the primary results and is updated to the same data freeze timepoint for SOC + DocP so is longer-term data than previously reported results for this arm [7].

The previously-reported comparisons of SOC + DocP versus SOC and SOC + AAP versus SOC had formal sample size calculations; there is no formal sample size calculation for this comparison: it is an opportunistic comparison between the contemporaneously recruited research arm patients. Although the recruitment overlap is only 17 months, 566 patients were allocated to the 2 research arms of interest and thus contribute substantial information to inform this comparison.

Standard survival analysis methods were used, following the approach for each of these underpinning comparisons; hazard ratios (HR) were estimated from adjusted Cox models, after checking that the proportional hazards assumption held, where an HR < 1 represents evidence in favour of SOC + AAP and HR > 1 represents evidence in favour of SOC + DocP. Nominal confidence intervals are presented at the 95% level. A *P*-value <0.1 was considered indicative of treatment-baseline characteristic interaction, recognising the limited power of the heterogeneity tests. Efficacy analyses were done in the intention-to-treatment basis, by allocated treatment. Safety analyses were done only in patients who started their allocated treatment.

## Results

### Accrual and characteristics

**Figure 2.**
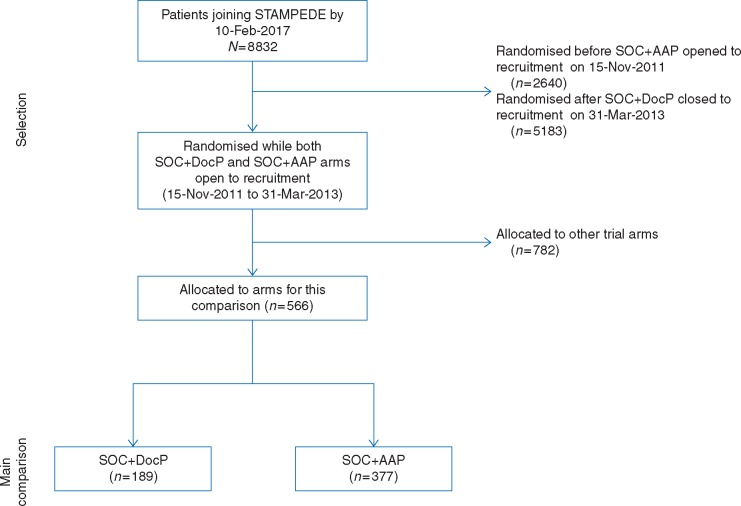
CONSORT diagram. SOC, standard-of-care; DocP, docetaxel+prednisolone/prednisone; AAP, abiraterone acetate+prednisolone/prednisone. Selection of patients for this comparison.

The dataset for this comparison was frozen on 10 February 2017. Between 15 November 2011 and 31 March 2013, 1348 patients joined all open arms STAMPEDE. Of the 566 randomised to the comparison reported here, 189 (14%) were allocated to SOC + DocP, 377 (28%) to SOC + AAP. The flow of patients to this comparison is shown in Figure 2. Table 1 shows the baseline characteristics of patients in this comparison which differ only slightly from the previous papers (summarised in supplementary Table S1, available at *Annals of Oncology* online). Median follow-up, calculated by reverse censoring on survival, was 48 months.

**Table 1.** Baseline characteristics of patients allocated to SOC + DocP or SOC + AAP by whether contributing to the direct comparison

	SOC + DocP		SOC + AAP		Overall	
Characteristic	*N*	%	*N*	%	*N*	%
Metastases						
M0	74	39	150	40	224	40
M1	115	61	227	60	342	60
Nodal stage						
N0	82	43	158	42	240	44
N+	99	52	202	53	301	56
NX	8	4	17	5	25	n/a
Combination						
N0 M0	43	23	84	22	127	22
N+M0	31	16	66	18	97	17
N0 M1	39	21	74	20	113	20
N+ M1	68	36	136	36	204	36
NX M1	8	4	17	5	25	4
Tumour category						
<T3	24	13	36	10	60	11
T3	123	65	249	66	372	69
T4	39	20	68	18	107	20
Tx	3	2	24	6	27	n/a
Gleason category						
≤7	35	19	91	25	126	23
8–10	153	81	276	75	429	76
Unknown	1	—	10	—	11	n/a
Previous local therapy						
No	183	97	350	93	533	94
Yes	6	3	27	7	33	6
WHO performance status						
0	149	79	300	80	449	79
1–2	40	21	77	20	117	21
Age (years)						
<70	134	71	267	71	401	71
70+	55	29	110	29	165	29
Median (quartiles)	66	(62–71)	66	(61–70)	66	(62–70)
Mean (SD)	66	(7)	66	(7)	66	(7)
Use of NSAID or aspirin						
No use	141	75	280	74	421	74
Uses either	48	25	97	26	145	26
PSA (ng/ml)						
Median (quartiles)	58	(29–162)	55	(20–194)	56	(22–185)
Mean (SD)	193	(421)	274	(631)	247	(571)
Ln PSA (ng/ml)						
Median (quartiles)	4.1	(3.4–5.1)	4.0	(3.0–5.3)	4.0	(3.1–5.2)
Mean (SD)	4.2	(1.4)	4.2	(1.6)	4.2	(1.5)
RT planned						
M0, yes	57	77	118	79	175	78
M0, no	17	23	32	21	49	22
M1, yes	12	10	21	9	33	10
M1, no	103	89	206	91	309	90
Hypertension						
Yes (still fit for trial)	64	34	149	40	213	38
No	125	66	227	60	352	62
Year of randomisation						
2011	15	8	27	7	42	7
2012	138	73	277	73	415	73
2013	36	19	73	19	109	19

### Overall survival

**Figure 3.**
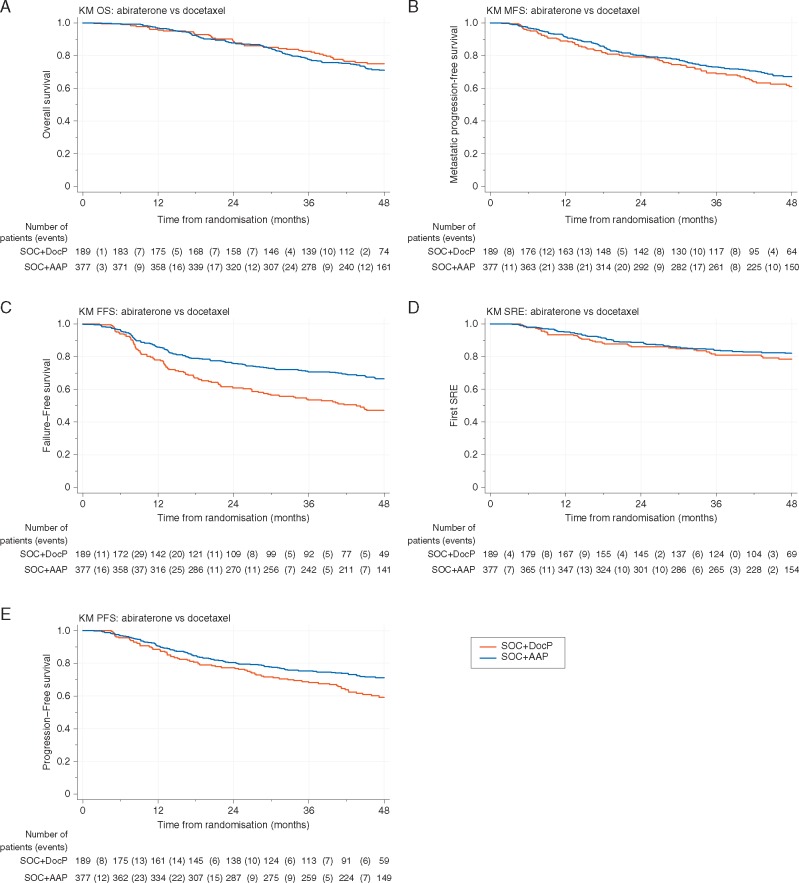
Efficacy analysis—survival, metastases-free survival, failure-free survival, skeletal-related events. Kaplan–Meier (survival) plots for the key efficacy outcome measures. Each step down the y-axis represents an event. The number of patients contributing information (at risk) over time since randomisation is shown under the table. The number of patients with an event between these points is shown in brackets. The number of patients censored in a time window is not shown, but is calculable as the difference between the number of patients at risk at two times points and the number of patients with events, e.g. in Figure 3E between 0 and 6 months on the SOC+AAP arm (377−362)−12=3 patients are censored.

There were 44/189 (23%) deaths on the SOC + DocP arm and 105/377 (28%) deaths on the SOC + AAP arm. The estimated HR = 1.16 (95% CI 0.82–1.65; ***P*** = 0.40) (Figure 3A). Estimates in patients with and without metastases are shown in Table 2, with HR = 1.51 (95% CI 0.58–3.93) in M0 patients and HR = 1.13 (95% CI 0.77–1.66) in M1 patients. There was no evidence of interaction in the treatment effect by baseline metastases (***P*** = 0.69).

**Table 2.** Hazard ratio for SOC + AAP relative to SOC + DocP from adjusted Cox models

Outcome measure	Patient group	Events/Pts SOC + DocP	Events/Pts SOC + AAP	Hazard ratio^a^ (95% CI)	*P*-value	Interaction by metastases *P*-value
Failure-free survival^b^						
	All	97/189	122/377	0.51 (0.39–0.67)	<0.001	

	M0	18/74	13/150	0.34 (0.16–0.69)	0.003	
	M1	79/115	109/227	0.56 (0.42–0.75)	<0.001	0.169
Progression-free survival^b^						
	All	72/189	103/377	0.65 (0.48–0.88)	0.005	

	M0	10/74	9/150	0.42 (0.17–1.05)	0.064	
	M1	62/115	94/227	0.69 (0.50–0.95)	0.023	0.323
Metastatic progression-free survival^c^						
	All	71/189	118/377	0.77 (0.57–1.03)	0.079	

	M0	10/74	18/150	0.91 (0.42–2.01)	0.824	
	M1	61/115	100/227	0.76 (0.55–1.04)	0.085	0.744
Freedom from symptomatic skeletal events						
	All	36/189	63/377	0.83 (0.55–1.25)	0.375	

	M0	2/74	5/150	1.28 (0.24–6.67)	0.771	
	M1	34/115	58/227	0.82 (0.53–1.25)	0.351	0.648
Overall survival						
	All	44/189	105/377	1.16 (0.82–1.65)	0.404	

	M0	6/74	16/150	1.51 (0.58–3.93)	0.395	
	M1	38/115	89/227	1.13 (0.77–1.66)	0.528	0.691

**Outcome measure**	**Patient group**	**Events/Pts SOC+Doc**	**Events/Pts SOC+AAP**	**Sub-hazard ratio**^d^**(95% CI)**	***P*-value**	**Interaction by metastases *P*-value**

Death from prostate cancer^e^						
	All	40/189	86/377	1.02 (0.70–1.49)	0.916	

	M0	4/74	6/150	0.82 (0.24–2.81)	0.751	
	M1	36/115	80/227	1.05 (0.71–1.56)	0.807	0.620
Death from other causes^f^						
	All	4/189	19/377	2.33 (0.77–6.99)	0.131	

	M0	2/74	10/150	3.00 (0.66–13.66)	0.155	
	M1	2/115	9/227	1.91 (0.43–8.41)	0.393	0.771

a From Cox proportional hazards model, adjusted for stratification factors at randomisation (except hospital and choice of hormone therapy) and stratified by time period.

b Includes death from prostate cancer.

c Includes death from any cause.

d From competing risks regression model, adjusted for stratification factors at randomisation (except hospital and choice of hormone therapy) and time period, and treating causes of death other than the focus as a competing event.

e Cause attributed on central death review; prostate cancer death as event, other cause of death as competing event.

f Cause attributed on central death review; other causes of death as event, prostate cancer as competing event.

Totally, 126/149 deaths were attributed to prostate cancer, comprising 10/22 and 116/127 deaths in patients with M0 and M1 disease at entry, respectively. Competing risks regression shows no evidence of a difference in prostate cancer-specific survival (sub-HR = 1.02, 95% CI 0.70–1.49). For non-prostate cancer-specific survival, with 23/149 deaths attributed to other causes, the sub-HR was 2.33 (95% CI 0.78–6.99). There was no evidence of heterogeneity of treatment effect by baseline metastases in either outcome.

### Other efficacy OMs

**Figure 4.**
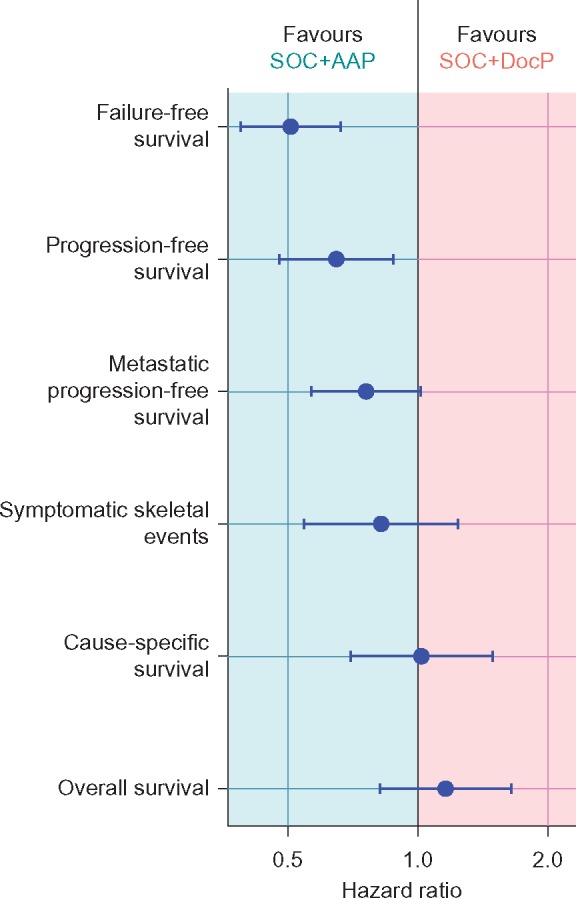
Depiction of disease state over time.

Table 2 shows the effect size overall and by whether the patients had metastases at entry for FFS, PFS, MPFS and skeletal-related events. There is no evidence of heterogeneity of the treatment effect by baseline metastases in any of these OMs. Figure 4 summarises the effect for all OMs.

### Safety

The safety population includes people who started their allocated treatment. While nearly all patients allocated to AAP started it, a proportion of those patients allocated to receive docetaxel declined to start it. Table 3 summarises the worst toxicity reported for patients over their time on trial in the safety population and shows differing patterns for adverse events according to treatment. The prevalence of grade 3 or 4 toxicity in patients with assessments at 1 year without a prior FFS event was 11% SOC + DocP and 11% SOC + AAP; at 2 years this was 11% SOC + DocP and 11% SOC + AAP.

**Table 3.** Worst adverse event (grade) reported over entire time on trial

	**SOC + Doc** (*n* = 189)	**SOC + AAP** (*n* = 377)
**Safety population**		
Number of patients included in analysis^a^	**172**	**373**
Patients with an adverse event—no. (%)		
Grade 1–5 adverse event	172 (100)	370 (99)
Grade 3–5 adverse event	86 (50)	180 (48)
Grade 3–5 adverse events—no. (%)		
Endocrine disorder	15 (9)	49 (13)
Febrile neutropenia	29 (17)	3 (1)
Neutropenia (neutrophils)	22 (13)	4 (1)
General disorder	18 (10)	21 (6)
Fatigue	7 (4)	8 (2)
Oedema	1 (1)	2 (1)
Musculoskeletal disorder	9 (5)	33 (9)
Cardiovascular disorder	6 (3)	32 (9)
Hypertension	0 (0)	12 (3)
Myocardial infarction	2 (1)	4 (1)
Cardiac dysrhythmia	1 (1)	5 (1)
Gastrointestinal disorder	9 (5)	28 (8)
Hepatic disorder	1 (1)	32 (9)
Increased AST	0 (0)	6 (2)
Increased ALT	1 (1)	23 (6)
Respiratory disorder	12 (7)	11 (3)
Dyspnoea	4 (2)	1 (1)
Renal disorder	5 (3)	20 (5)
Lab abnormalities	9 (5)	11 (3)
Hypokalaemia	0 (0)	3 (1)

a The safety population includes patients who started their allocated treatment.

### Second-line treatment

**Figure 5.**
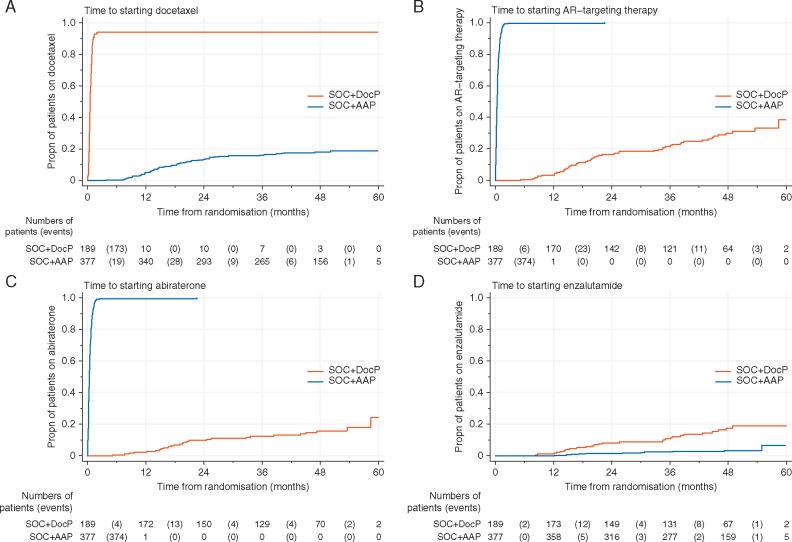
Time from randomisation to reported starting docetaxel, AAP, enzalutamide or AR-targeting therapy. Kaplan–Meier (survival) plots showing cumulative incidence of exposure to treatments after randomisation. Each step up the *y*-axis represents an event, namely starting that particular treatment. The number of patients contributing information (at risk) over time since randomisation is shown under the table. The number of patients with an event between these points is shown in brackets. For example, in Figure 4C between 24 and 36 months after randomisation, 4 patients on the SOC+DocP arm report starting abiraterone and (150−129)−4 are 17 are censored and may start in the future.

**Figure 6.**
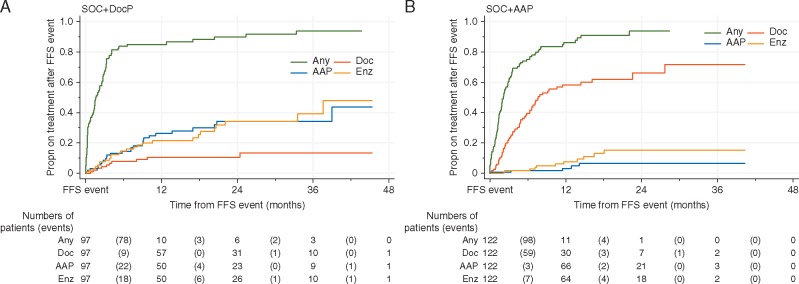
Time from failure-free survival event to subsequent treatment by allocated treatment. Kaplan–Meier (survival) plots showing cumulative incidence of exposure to treatments after a failure-free survival (FFS) event. Doc, docetaxel; AAP, abiraterone acetate + prednisolone; Enz, enzalutamide. Each step up the *y*-axis represents an event, namely starting that particular treatment.

Figure 5 shows time from randomisation to any subsequent exposure to docetaxel or AR-targeted therapy with AAP or enzalutamide. Figure 6 shows time from an FFS event to reported exposure to selected treatments that are licensed for CRPC: docetaxel, AAP, enzalutamide. There was limited reported use of cabazitaxel, radium and sipuleucel-T at this point (not shown).

## Discussion

We and others have previously shown a survival advantage for adding docetaxel (with or without prednisolone/prednisone) and for adding abiraterone acetate and prednisolone/prednisone, in patients starting long-term hormone therapy for the first time [4–11]. However, there is currently no direct evidence available to help clinicians or patients assess which combination might be better. Here, we reported a pre-specified (but not pre-powered) analysis using only patients who were randomised during a period of the study when recruitment to the two research arms overlapped. We used data collected prospectively from over 100 sites across two countries as part of a clinical trial protocol. The MAMS platform design of STAMPEDE, an approach sometimes referred to as a master protocol [16], facilitated this comparison. Separate, traditional, two-arm RCTs, would not have allowed any directly randomised comparative evidence to be available so soon.

Our recently reported overall treatment effect on survival, in STAMPEDE, for adding AAP compared with the SOC (HR = 0.63) [10] was larger than the previously-reported overall treatment effect, in STAMPEDE, on survival for adding DocP to the same SOC (HR = 0.78) [7]. The earlier secondary efficacy OMs favoured adding AAP over DocP, including FFS—perhaps unsurprising given the direct antiandrogenic action of AAP (around four in every five FFS events was driven only by a rise in PSA) and PFS (which excludes rising PSA). There was weak evidence favouring AAP for MPFS and no evidence of a difference in symptomatic skeletal events, prostate cancer-specific survival or OS.

Comparing the results indirectly of these two therapies by readers extracting data from STAMPEDE’s AAP and docetaxel papers [7, 10] may not be the most appropriate way to compare the relative effectiveness: the patient cohorts were all not randomised contemporaneously and there may be confounding biases when comparing the two datasets, in particular, many DocP patients had very limited salvage CRPC options compared with AAP patients, simply due to the timing of licences of new therapies (see below).

Importantly, the two therapies are being used in different ways. AAP is used until the patient has castrate-resistant prostate cancer (CRPC), often lasting many years and consequently exhausting a major therapy option for CRPC. In contrast, DocP is given as an 18-week course thus all CRPC options should remain available. Our data reveal important differences in the pattern of treatment failure yet we do not see any differences in survival, suggesting that the relative time spent before and after first-line treatment failure are quite different by initial treatment. This may explain why the early, often biochemically driven OMs, favour AAP but the later post CRPC end points such as skeletal events, prostate cancer-specific survival and OS show no good evidence of a difference. Men receiving DocP will thus spend longer with CRPC than men receiving AAP but with a broader range of more effective options available. Supplementary Figure S1, available at *Annals of Oncology* online, shows the status of all patients at each moment in time after randomisation. That the DocP cohort had more durable survival after failure, perhaps longer than before failure, may be important in counselling patients’ biochemically failing after DocP.

The number of events is an important consideration in time-to-event analyses. The number of patients with metastases at baseline was balanced by arm, but, particularly because of their poorer prognosis, these patients tend to predominate in this analysis. There is no evidence of heterogeneity in the treatment effect by baseline metastasis for any of the OMs, but power to detect any heterogeneity is very limited, especially in later OMs with fewer events.

The patterns of toxicity are quite different for the two treatment approaches, consistent with the known effects of the drugs. The proportion of patients reporting at least one grade 3 or worse toxicity was similar and in line with previously reported toxicities for these agents (Table 3). In patients who started their allocated treatment and who are without disease progression at 1 year, the prevalence of grade 3 or worse toxicity was about 11% on both arms and very similar to our previous estimate for SOC. Nearly all patients started their allocated abiraterone, whereas about 1 in 12 patients did not start their allocated docetaxel. Our results may change future compliance with both treatments in routine practice; but the lack of compliance with allocated treatment of docetaxel is likely to have had some impact on our estimated effect sizes.

A key limitation is that the comparison was opportunistic and not designed in the usual way, hence power is limited to detect any realistic differences. The trigger for the analysis was the reporting of our ‘abiraterone comparison’ data [10]. The unequal allocation ratio reflects the planned design of the comparisons. The allocated treatment being given was not masked for practical reasons. This, of course, allowed for relapse therapies to be given at the investigator’s discretion. We observed that after relapse, many patients received the treatment class that they had not received up-front.

Salvage options have changed over time: men recruited earlier on to DocP (2005–2013) will have had very different options to those recruited later to AAP (2011–2014) when there were more CRPC therapies likely available, including AAP [17, 18], cabazitaxel [19], docetaxel [20, 21], enzalutamide [22, 23], radium-223 [24] and sipuleucel-T [25] (although not widely accessible in Europe). For this analysis, we limited ourselves to patients contemporaneously randomised to either arm to make this comparison as fair as possible. However, FFS events generally happened sooner with DocP than with AAP in time from randomisation and, therefore, calendar year (Table 4) may partially influence outcomes. Furthermore, a FFS event was more of an indication to change treatments on DocP; AAP continued beyond this point.

**Table 4.** Year of FFS event and death by arm

Year of event	FFS event				Death			
	SOC + DocP		SOC + AAP		SOC + DocP		SOC + AAP	
	*N*	%	*N*	%	*N*	%	*N*	%
2012	14	7	25	6	1	1	5	1
2013	38	20	43	11	12	6	18	5
2014	25	13	33	9	9	5	33	9
2015	14	7	11	3	16	8	38	10
2016	6	3	10	3	6	3	11	3
No event	92	49	255	68	145	77	272	72

As far as we are aware there are no ongoing randomised trials directly comparing adding AAP versus adding docetaxel for patients starting long-term ADT. All of our published STAMPEDE data have contributed to the STOpCaP aggregate data network meta-analysis that has used all of the reported RCTs in metastatic patients to perform indirect comparisons and allow some assessment of potential ranking of effective therapies. This aggregate data analysis (co-submitted) will be supplemented by a forthcoming individual patient data (IPD) network meta-analysis which will hopefully provide a more accurate reflection of the temporal interval between the application of the two different therapies, to which STAMPEDE will contribute all relevant data. We will continue to follow-up patients for long-term OMs.

Considering their mechanisms of action and their proven oncological benefits, the question is raised of whether a combination of AAP plus docetaxel might lead to an approximately additive benefit of using them both, further extending survival. Randomised data on docetaxel with or without abiraterone will emerge from a subset the PEACE-1 trial (https://clinicaltrials.gov/ct2/show/NCT01957436), as will non-randomised, time-stratified data on abiraterone with or without docetaxel. Similarly comparative data will also emerge for enzalutamide, another AR-targeted therapy, from the ENZAMET trial (https://clinicaltrials.gov/ct2/show/NCT02446405) and with the combination of enzalutamide and AAP in STAMPEDE (Figure 1). 

In conclusion, there are now two systemic therapies, DocP and AAP, which have shown a survival benefit from RCTs when added to treatment of patients starting long-term ADT for the first time. The evidence from our directly randomised data comparing these two therapies showed no evidence of a difference in overall or prostate cancer-specific survival, nor in other important outcomes such as symptomatic skeletal events, suggesting that both currently remain viable new standards-of-care.

## Supplementary Material

Supplementary Data

## Trials Unit Staff (from 2011 onwards)

### MRC Clinical Trials Unit at UCL

- **Statisticians**—Matthew Sydes, Max Parmar, Melissa Spears, Chris Brawley; *Previously—*Gordana Jovic, Rachel Jinks, Patrick Royston, Sophie Barthel, Babak Choodari-Oskooei, Daniel Bratton, Andrew Embleton
- **Project and Trial Managers**—Claire Amos, Nafisah Atako; Claire Murphy, Joanna Calvert, Mazna Anjum, Chris Wanstall, Arlen Wilcox; *Previously—*Sharon Naylor, Neil Kelk, James Latham, Jacqui Nuttall, Karen Sanders, Tom Fairfield, Charlene Green, Francesca Schiavone, Katie Ward, Mazna Anjum, Anna Herasimtschuk, Jenny Petrie, Alanna Brown, Orla Prendiville
- **Data Managers**—Carly Au, Danielle Johnson, Lina Bergstrom, Tasmin Philips; *Previously—*Emma Donoghue, Tim Smith, Jacque Millett, Shama Hassan, Philip Pollock, Richard Gracie, Laura Van Dyck, Charlene Green, Elizabeth Clark, Sara Peres, Hannah Gardner, Dominic Hague, Katie Ward, Peter Vaughan, Eva Ades, Hannah Babiker, Zohrah Khan, Nargis Begum, Saba Khan, Jenna Grabey
- **Data Scientists and Programmers**—Nadine Van Looy, Zaheer Islam, Dominic Hague; *Previously—*Lindsey Masters, Will Cragg, Sajad Khan
- **Clinicians**—Clare Gilson, Alastair Ritchie; *Previously—*Sarah Meredith, Ruth Langley
- **Trial Assistants**—Stephanie Wetton, Amy Fiddament; *Previously—*Leigh Dobson, Alexandra Wadia, Nat Thorogood, Shanaz, Sohail, Tracey Fisher, Andrew Whitney

**Swiss Group for Cancer Clinical Research**

- **Project and Trial Managers**—Corinne Schar; *Previously—*Estelle Cassolly
- **Patient and Public Involvement representatives**—David Matheson, Robin Millman
