# Supplementary figures and images for: Adding abiraterone or docetaxel to long-term hormone therapy for prostate cancer: directly randomised data from the STAMPEDE multi-arm, multi-stage platform protocol
Source: Ann Oncol. 2018 Feb 26;29(5):1235–48. doi: 10.1093/annonc/mdy072 (PMC5961425; doi:10.1093/annonc/mdy072)

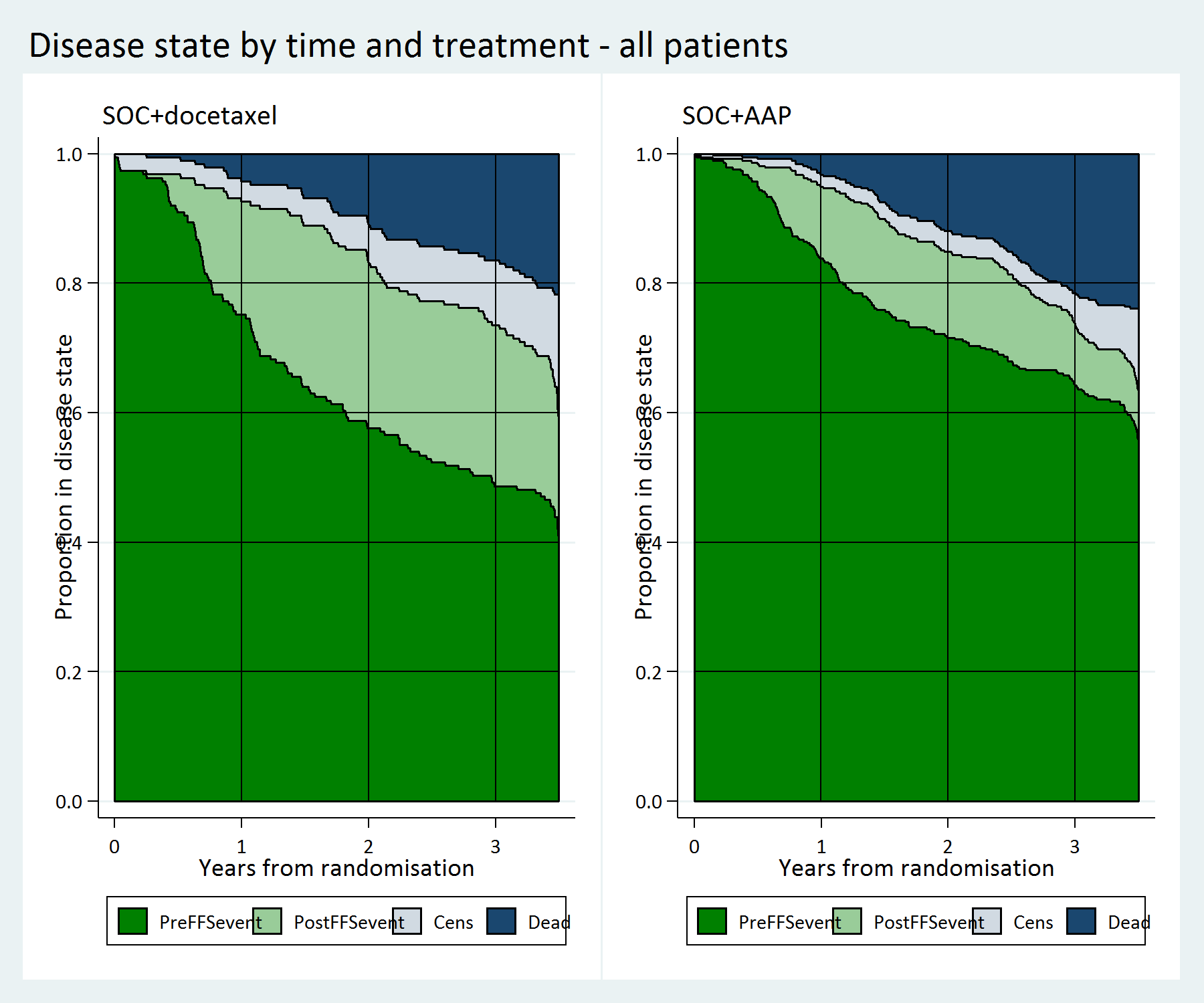

Supplement: Supplementary Data [file mdy072_supp.zip › mdy072-suppl_data/Supp_Figure_1a.png]

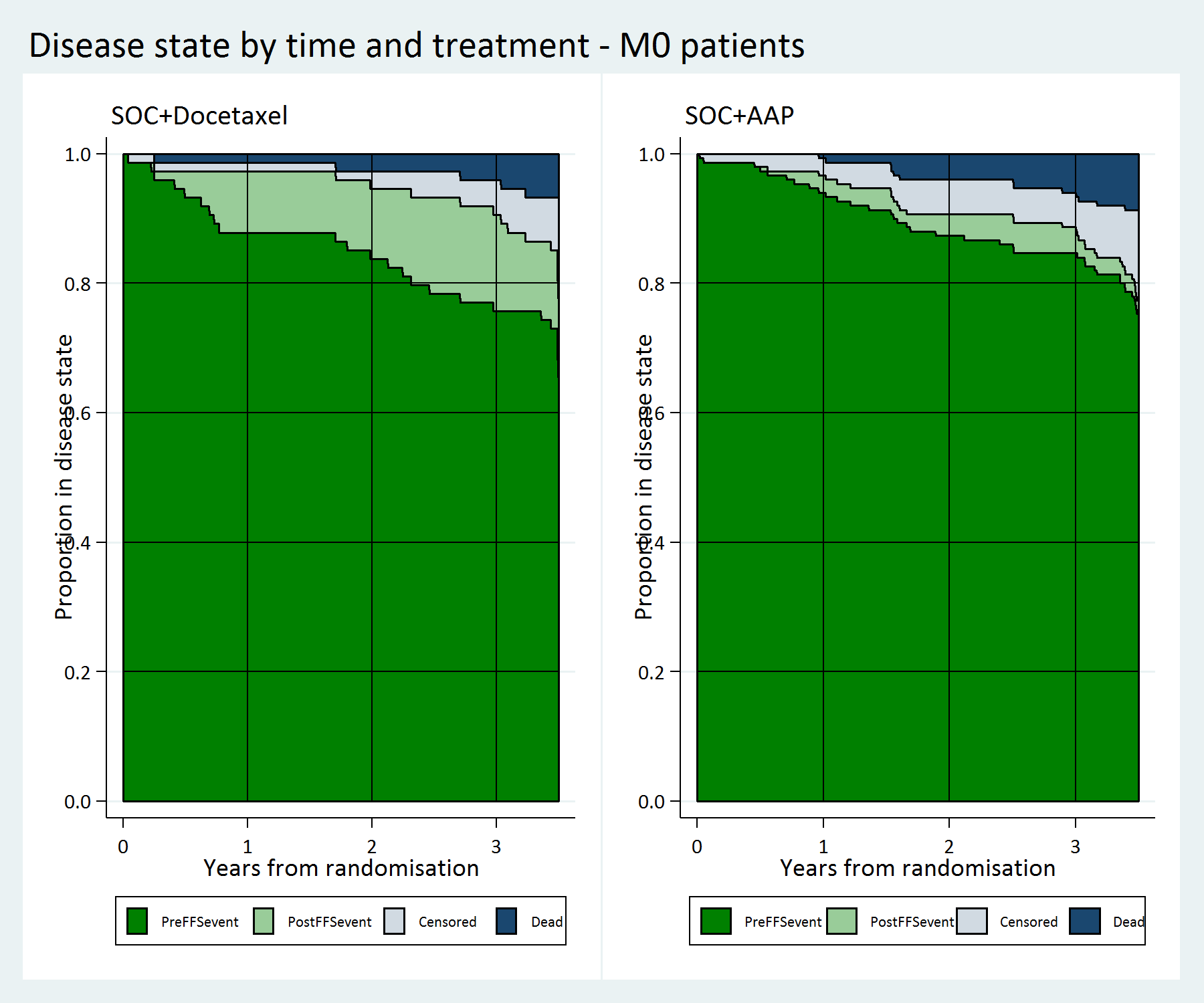

Supplement: Supplementary Data [file mdy072_supp.zip › mdy072-suppl_data/Supp_Figure_1b.png]

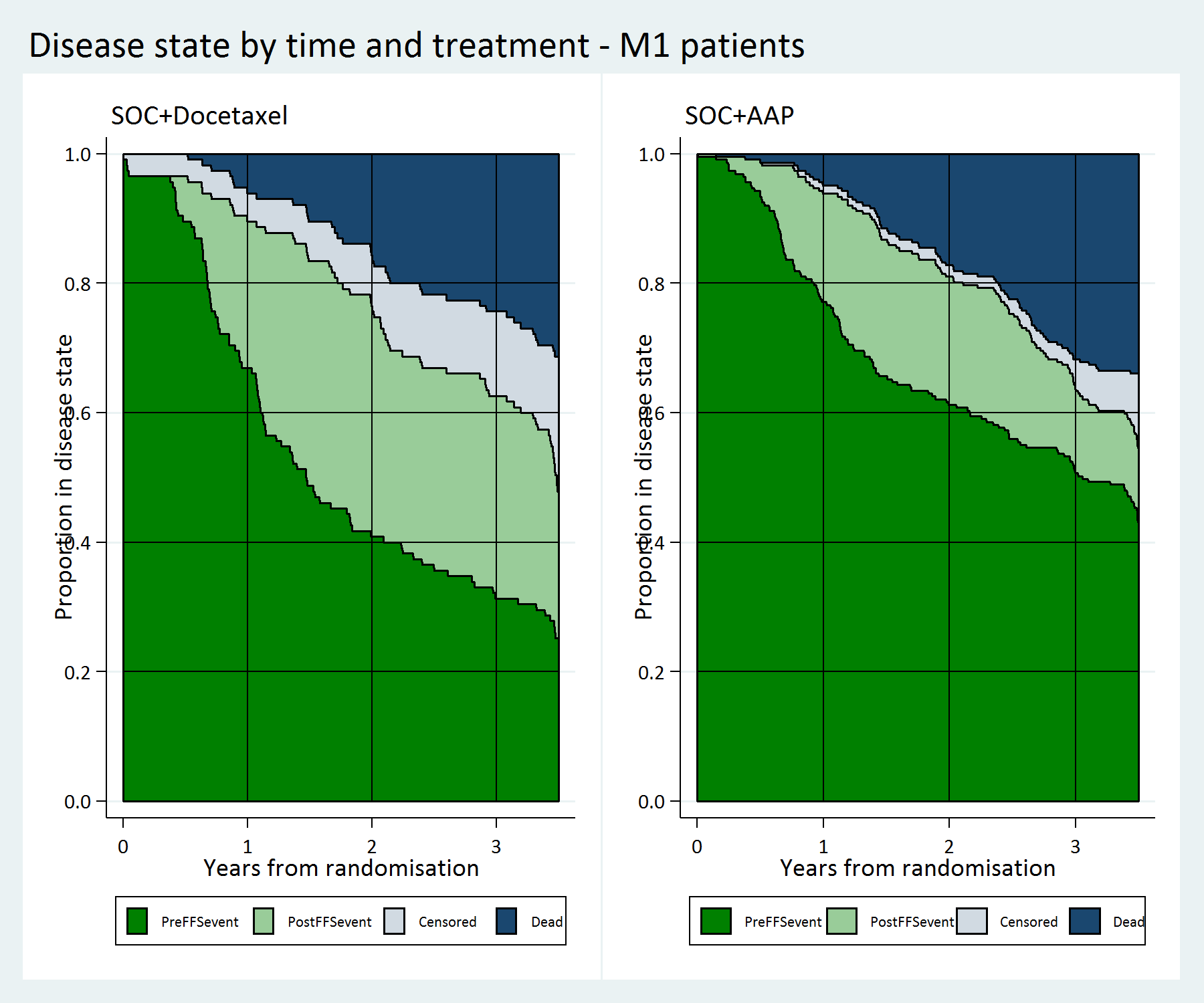

Supplement: Supplementary Data [file mdy072_supp.zip › mdy072-suppl_data/Supp_Figure_1c.png]
